# United Kingdom value set for the functional assessment of cancer therapy eight dimension (FACT-8D) preference-based quality of life instrument

**DOI:** 10.1007/s10198-025-01844-w

**Published:** 2025-10-08

**Authors:** R. Norman, R. Campbell, D. Rowen, R. Viney, D. J. Street, F. Müller, R. Mercieca-Bebber, A. S. Pickard, D. Cella, J. W. Shaw, M. T. King

**Affiliations:** 1 School of Population Health, Curtin University, Perth, WA Australia; 2 University of Sydney, School of Psychology, Sydney, NSW Australia; 3 Sheffield Centre for Health and Related Research, University of Sheffield, Sheffield, South Yorkshire UK; 4 Centre for Health Economics Research & Evaluation, Faculty of Health, University of Technology Sydney, Sydney, NSW Australia; 5 Amsterdam UMC, University of Amsterdam, Medical Psychology, Amsterdam, the Netherlands; 6 University of Sydney, Faculty of Medicine and Health, NHMRC Clinical Trials Centre, Sydney, NSW Australia; 7 Department of Pharmacy Systems, Outcomes and Policy, College of Pharmacy, University of Illinois at Chicago, Chicago, IL USA; 8 Department of Medical Social Sciences, Northwestern University Feinberg School of Medicine, Chicago, IL USA; 9 Patient-Reported Outcomes Assessment, Worldwide Health Economics and Outcomes Research, Bristol Myers Squibb, Lawrenceville, NJ USA

**Keywords:** Condition-specific, Cancer-specific, Oncology, Preference-based, Value set, Multi-attribute utility, Quality of life, Health-related quality of life, Quality adjusted life year, QALY

## Abstract

**Objectives:**

To develop a value set reflecting the United Kingdom (UK) general population’s preferences for health states described by the Functional Assessment of Cancer Therapy (FACT) eight-Dimension preference-based multi-attribute utility instrument (FACT-8D), derived from the FACT-General (FACT-G) cancer-specific health-related quality-of-life (HRQL) questionnaire.

**Methods:**

A UK online panel was quota-sampled to achieve a general population sample representative by sex and age (≥ 18y). A discrete choice experiment (DCE) was used to value health states. The valuation task involved choosing between pairs of health states (choice-sets) described by varying levels of the FACT-8D HRQL dimensions and survival (life-years). The DCE included 800 choice-sets; each respondent was randomly allocated 16 choice-sets. Data were analyzed using conditional logistic regression parameterized to fit the quality-adjusted life-year framework, weighted for sociodemographic variables that were non-representative of the UK general population. Preference weights were calculated as the ratio of HRQL-level coefficients to the survival coefficient.

**Results:**

2239 panel members opted in, 2125 (95%) completed at least one choice-set, and 2054 (92%) completed 16 choice-sets. Pain and nausea were associated with the largest utility weights, followed by problems with work and sadness. Within dimensions, more severe HRQL levels were generally associated with larger decrements. A preference-weighting algorithm to estimate UK utilities from responses to the FACT-General questionnaire was generated. The worst health state’s value was -0.402, worse than dead.

**Conclusions:**

This value set provides UK population utilities for health states defined by the FACT-8D for use in evaluating oncology treatments.

**Supplementary Information:**

The online version contains supplementary material available at 10.1007/s10198-025-01844-w.

## Highlights

The FACT-G is a widely used tool for measuring health in cancer settings, and this work provides a value set to allow UK values to be applied to FACT-G data in cost-utility analysis.

The FACT-8D has been shown to be sensitive and responsive to health in oncology; having this value set potentially allows a more accurate characterisation of health change.

While UK decision makers prefer generic instruments, we believe our findings can be useful, both in settings where generic instruments might be too insensitive to change, or as part of sensitivity analysis.

**Supplementary Information:**

The online version contains supplementary material available at 10.1007/s10198-025-01844-w.

## Introduction

Economic evaluation of new health care therapies and technologies is central to decision making in many countries, including the United Kingdom (UK), for example through the National Institute for Health and Care Excellence (NICE) [1] and the Scottish Medicines Consortium (SMC) [2]. Cost-utility analysis (CUA) is a policy-relevant and valuable form of economic evaluation because it quantifies health outcomes on a metric that is applicable across health conditions. In CUA, the quality-adjusted life year (QALY) is a widely used metric of health that captures changes in both morbidity and mortality. The quality adjustment metric used to calculate QALYs is a utility index (or utility weight), with a maximum of one representing full health, zero representing being dead, and negative values representing health states worse than dead [3]. The quality adjustment metric is usually generated using preference-based measures (PBMs) such as the generic EQ-5D [4] or SF-6D [5, 6]. These have two components: a descriptive system that systematically describes a comprehensive set of health states (also referred to as a ‘health state classification system’), and a set of utility weights (also referred to as ‘preference weights’) generated using a valuation survey that elicits preferences, conventionally from a general population sample to yield societal preference weights. Typically, an algorithm specifies how these utility weights are used to produce a utility score for every health state in the classification system, which can then be used to generate QALYs.

PBMs can either be developed *de novo* or by adapting pre-existing health-related quality of life (HRQL) profile measures [7, 8]. The advantage of the latter approach is that it means utility weights can be derived using data that are already collected using an existing measure without additional respondent burden. Such an approach has been applied to the Functional Assessment of Cancer Therapy General (FACT-G), a widely-used HRQL questionnaire for people with cancer [9]. The Functional Assessment of Cancer Therapy Eight Dimension (FACT-8D) [10] is a multi-attribute utility instrument (MAUI) derived from the FACT-G, which is cancer-specific HRQL profile measure, i.e. it captures a person’s self-reported HRQL and summarises it as a profile of domain scores [11]. The FACT-G is widely used in oncology clinical trials [12]. The Multi-Attribute Utility in Cancer (MAUCa) Consortium is a group of international researchers who have initiated a series of country-specific value sets for the FACT-8D. The FACT-8D enables quantification of utility scores from FACT-G data and captures dimensions reflecting symptoms and impacts of cancer and its treatments that are not typically included in generic instruments; specifically nausea, fatigue, sleep problems and worry about future health [10]. In a health technology assessment context, these inclusions are important as they directly ask patients about all relevant domains affecting their health as a result of their disease and treatment. As these domains are not explicitly captured in most generic instruments, the FACT-8D has the potential to address limitations of existing PBMs, generating more reliable QALY estimates while also minimising onerous data reporting requirements for participants through using a widely-used quality of life instrument to estimate utility weights rather than needing a separate, stand-alone utility instrument. While mapping algorithms are available to predict utilities from FACT-G responses via mapping to generic MAUIs [13], the FACT-8D may be considered theoretically and empirically stronger because it directly generates utilities and comprises a descriptive system and a valuation method that complies with the Checklist for Reporting Valuation Studies of Multi-Attribute Utility-Based Instruments [14]. Regarding the measurement properties of the FACT-8D, this remains an area where additional work is required. Regarding the QLU-C10D, derived from the EORTC QLQ-C30 using a similar approach to the FACT-8D, recent evidence has suggested that QLU-C10D has appropriate psychometric properties and higher statistical efficiency than generic PBMs in cancer [15], but that it may be less sensitive to performance status groups in a glioblastoma cohort [16].

The FACT-8D descriptive system has been valued in an Australian general population sample [10]. However, preferences for health may differ across countries [17, 18]. Such differences may reflect underlying cultural, social and work differences across countries, different sociodemographic profiles of valuation samples [19], and artefactual differences caused by use of a range of elicitation techniques and study protocols [20]. Many international agencies specify preferred methods for health technology assessment submissions to inform resource allocation decisions. Several countries require country-specific utility weights [21]. NICE in particular requires UK weights, and further specifies that the utility weights should be elicited using a representative sample of the general population [22]. It is important that any real between-country differences in general population preferences be reflected in the utility algorithm used for health technology assessment (HTA) in a particular country, so country-specific preference weights need to be developed. To avoid artefactual confounding across countries, the MAUCa Consortium derived a standard method for valuing the FACT-8D in general population samples, described below. Using this method, we have estimated value sets in Australia [10], Canada [23], and the United States [24]. The aim of this current paper is to apply this valuation method in a UK general population sample to produce the first UK-specific utility weights for the FACT-8D, thus facilitating the estimation of utility weights in a way that captures the typical health-related quality of life effects of cancer and cancer therapy.

## Methods

This research was conceived, designed and conducted by the MAUCa Consortium. The [REDCATED TO PREVENT UNBLINDING] Human Research Ethics Committee approved MAUCa’s program of research (No. 13207). The UK valuation study was deemed exempt from ethics review by the National Health Service Health Research Authority.

### Survey sampling and implementation

A cross-sectional population-based survey conducted from 24^th^ September to 5th November 2020 collected sociodemographic and health status data, with a discrete choice experiment (DCE) included as the valuation component. SurveyEngine, a company specializing in online choice experiments, managed sample recruitment (via a UK online panel), survey administration and data collection. SurveyEngine and its panel provider complied with the International Code on Market, Opinion and Social Research and Data Analytics [25]. Members of the online panel were eligible if 18 years or older and able to read English. Online panelists received an email invitation, including a hyperlink to the survey. Any who attempted to enter the survey via mobile phones were excluded as the DCE was too complex for a small screen. Consent was sought from those who successfully entered the survey, and those who consented were screened for quota sampling to ensure that the age and sex distributions of the sample approximated those of the UK general population. Those who completed the survey were awarded panel points to accumulate with other surveys they complete. All data sent to the research team were anonymous and the demographic data were designed to not allow any possible re-identification. Data were stored on secure University servers, and are protected according to ISO 27001 data standards.

### Discrete choice experiment

A DCE was used to generate preference data that were used to estimate the parameters of the UK FACT-8D preference-weighting algorithm. The DCE contained nine attributes: survival duration and the FACT-8D dimensions (pain, fatigue, nausea, sleep, work, support, sadness, worry). Table 1 shows how the HRQL attributes and levels in the DCE mapped to the FACT-8D descriptive system and corresponding FACT-G source items. Previous work demonstrated that DCE respondents found choice tasks with both positively- and negatively-worded items very difficult to interpret, leading to large regression coefficients in unexpected directions (10). Therefore, the FACT-8D reverses some items as shown in Table 1, so all items are expressed negatively. In the FACT-8D descriptive system, each HRQL dimension has five levels, corresponding to the five response options in the FACT-G source items; the FACT-8D therefore describes over 390,000 possible health states (5^8^ = 390,625). In the DCE, survival duration had four levels: 1, 2, 5 and 10 years.

**Table 1:** Mapping between the FACT-8D descriptive system (dimensions and levels), FACT-G items, and attributes and levels in the discrete choice experiment

FACT-8D dimension	DCE attribute wording	FACT-8D level	Descriptor	FACT-G item^a^ scores^b^
Pain	Pain	1	Not at all	GP4 = 0
		2	A little bit	GP4 = 1
		3	Somewhat	GP4 = 2
		4	Quite a bit	GP4 = 3
		5	Very much	GP4 = 4
Fatigue	Fatigue	1	Not at all	GP1 = 0
		2	A little bit	GP1 = 1
		3	Somewhat	GP1 = 2
		4	Quite a bit	GP1 = 3
		5	Very much	GP1 = 4
Nausea	Nausea	1	Not at all	GP2 = 0
		2	A little bit	GP2 = 1
		3	Somewhat	GP2 = 2
		4	Quite a bit	GP2 = 3
		5	Very much	GP2 = 4
Sleep^c^	Problems sleeping	1	Not at all	GF5 = 4
		2	A little bit	GF5 = 3
		3	Somewhat	GF5 = 2
		4	Quite a bit	GF5 = 1
		5	Very much	GF5 = 0
Work^c^	Problems doing work including work at home	1	Not at all	GF1 = 4
		2	A little bit	GF1 = 3
		3	Somewhat	GF1 = 2
		4	Quite a bit	GF1 = 1
		5	Very much	GF1 = 0
Support^c,d^	Problems with support from my family and/or friends	1	Not at all	max(GS2, GS3) = 4
		2	A little bit	max(GS2, GS3) = 3
		3	Somewhat	max(GS2, GS3) = 2
		4	Quite a bit	max(GS2, GS3) = 1
		5	Very much	max(GS2, GS3) = 0
Sadness	Sadness	1	Not at all	GE1 = 0
		2	A little bit	GE1 = 1
		3	Somewhat	GE1 = 2
		4	Quite a bit	GE1 = 3
		5	Very much	GE1 = 4
Worry my health will get worse	Worry my health will get worse	1	Not at all	GE6 = 0
		2	A little bit	GE6 = 1
		3	Somewhat	GE6 = 2
		4	Quite a bit	GE6 = 3
		5	Very much	GE6 = 4

a. Nine FACT-G items are included in the FACT-8D: GP4 (I have pain), GP1 (I have a lack of energy), GP2 (I have nausea), GF5 (I am sleeping well), GF1 (I am able to work including work at home), GS2 (I get emotional support from my family) and GS3 (I get support from my friends), GE1 (I feel sad), GE6 (I worry that my condition will get worse)

b. FACT-G item scores correspond to the following response options: 0 (Not at all), 1 (A little bit), 2 (Somewhat), 3 (Quite a bit), 4 (Very much)

c. Because the FACT-G items that determine the FACT-8D dimensions Sleep, Work, Support are positively framed, reverse scoring is required so that FACT-8D Level 0 represents the best score and Level 4 represents the worst score across all dimensions

d. The FACT-8D Support dimension contains two items; the FACT-8D level allocated is the maximum score of the FACT-G items GS2 and GS3, i.e. the best level of support, whether from family or friends

The DCE experimental design comprised 800 choice-sets that optimized statistical efficiency in estimating the utility model parameters assuming a null prior. Each choice-set comprised two FACT-8D health states, each described by nine attributes (eight HRQL dimensions and duration). We simplified the cognitive task by constraining the number of attributes that differed between health states in any choice-set to five, in line with a typical number of attributes in DCEs used to develop preference-based measure value sets [26]. The design was developed using a generator approach [27]. For any generator-developed design an initial design and a set of generators are required. In this case, since the instrument has 8 dimensions each with 5 levels, we started with the first 8 columns of a publicly available 50 run orthogonal array [28]. As we wanted to be able to estimate an interaction between duration and each of the dimensions of the instrument, we appended each of the possible durations to this giving a total of 200 runs in the initial design used to generate the choice sets. To reduce task complexity, we decided to have only 5 of the 9 items different between the options in each choice set; that is, we decided to have the options in each choice set overlap on 4 of the dimensions. This means that every generator needs to have 4 zero entries. If we wanted to be able to ensure that every pair of attributes was overlapped together the same number of times we would need 18 generators and this was clearly impractical. Instead we elected to find a set of 4 generators, and hence 800 choice sets, in which each attribute was overlapped in 200 (two attributes) or 400 (7 attributes) of the choice sets. Each respondent was then randomly allocated 16 pairs (without replacement).

**Fig. 1:**
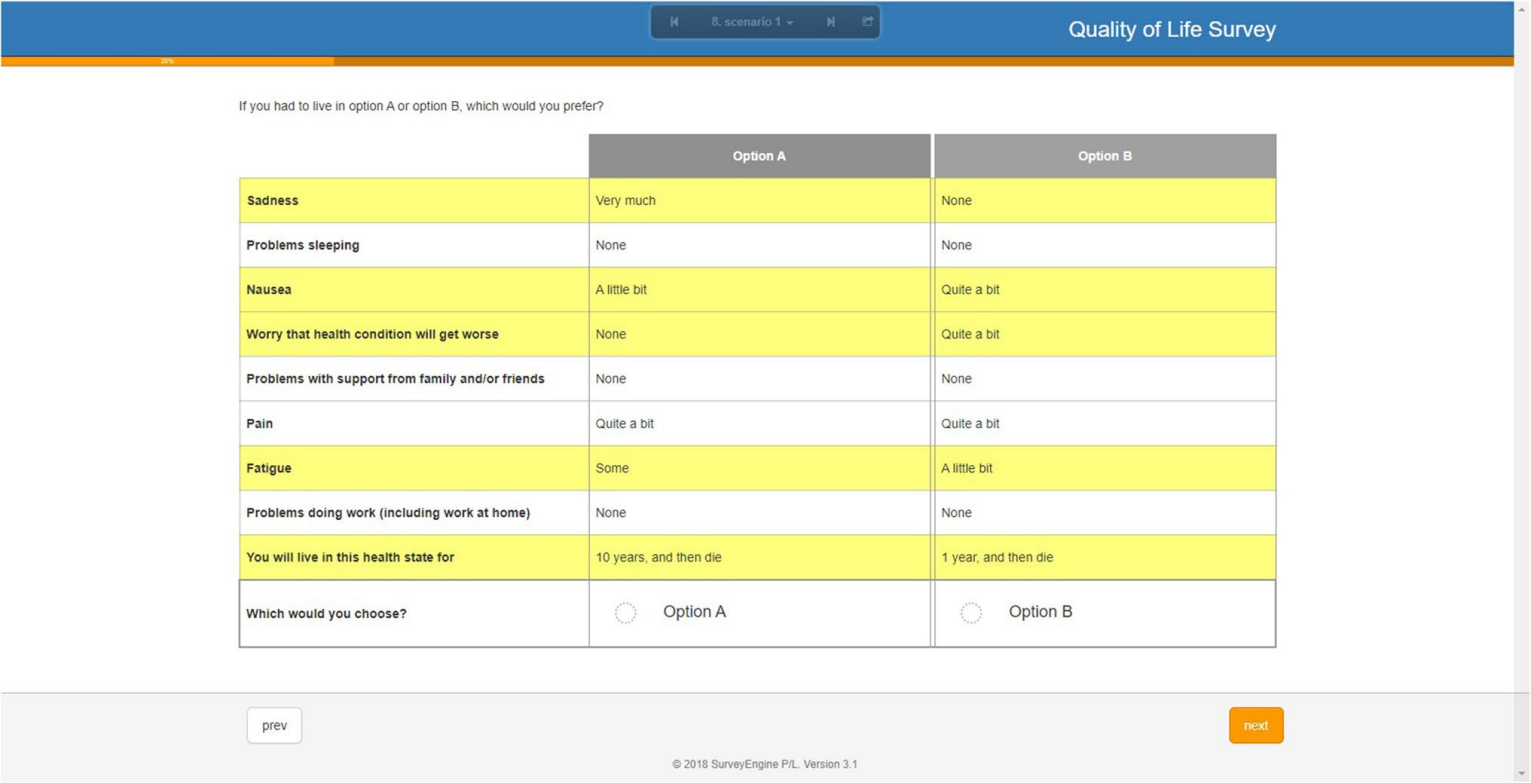
An example choice set

The valuation task required participants to consider 16 pairs of hypothetical health states (i.e., 16 choice-sets), described as ‘Option A’ and ‘Option B’ (Fig. 1), and for each choice-set, select the health state they would prefer to live in until death. Dimensions that differed between Options A and B were highlighted in yellow, a presentation format preferred by participants in our previous DCE valuation methods experiment [29]. Prior testing of this format for the FACT-8D has previously been reported in Australia, and led to considerable wording changes to support interpretability [10].

There were two levels of randomization in the DCE component of the survey: 1) each respondent was allocated to a block of 16 choice-sets from the 800 in the DCE design; and 2) which option was seen as Option A or Option B was randomized within each choice-set. The nine DCE attributes were always presented in the same order, as previous work showed that order does not systematically bias preference weights [30].

### Other survey content

**Fig. 2:**
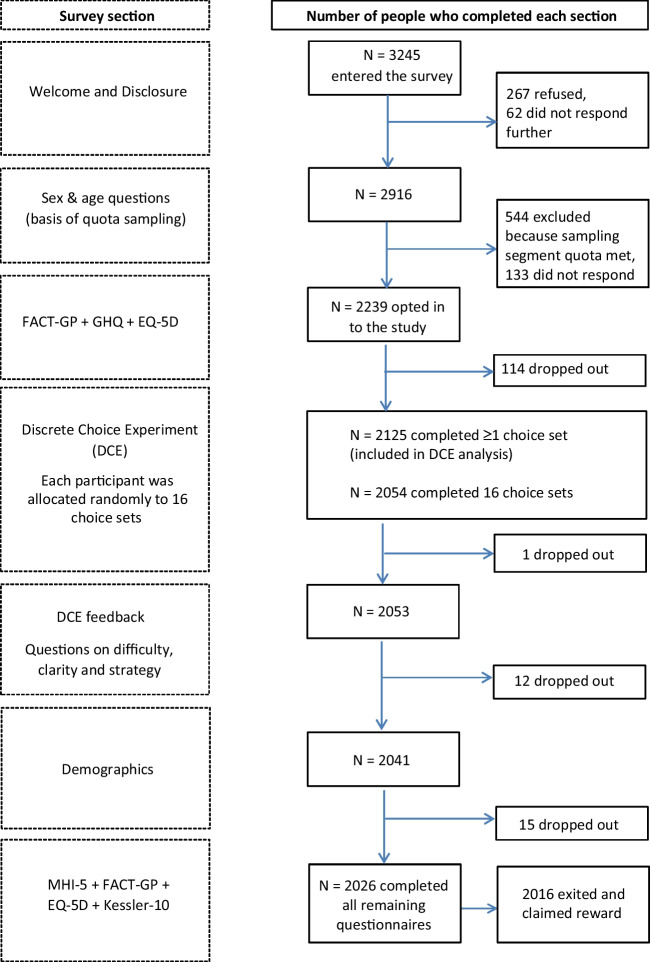
Respondent flow and sample sizes for each component of the UK FACT-8D valuation survey

The survey included sociodemographic characteristics, the FACT-G [11] and self-reported general health. The order of survey components is shown in Fig. 2. After completing the DCE component, participants were asked four fixed-format questions about the difficulty and clarity of the valuation task and strategy used to choose between health states (supplementary Appendix A).

### Statistical analyses

Descriptive statistics summarized sample demographics, self-reported general health and participant feedback on the DCE valuation task. Sample representativeness was assessed against UK population reference data for demographics and self-reported general health using chi-square tests.

DCE data quality was assessed by tallying how many respondents chose either all A’s or all B’s across the choice-sets and by considering the time respondents took to complete the survey. We divided respondents into deciles based on total survey time, ran a conditional logit on the DCE data in each decile, then graphed the Pseudo-R^2^ and the number of statistically significant coefficients for each decile, interpreting low values on either indicator as suggesting relatively low quality data.

The DCE data were analyzed with STATA 13.0 [31], using a functional form used previously to estimate utilities from DCE data consistent with standard QALY model restrictions [32–36]. The QALY model requires a health utility scale where dead anchors the scale at zero, i.e., ‘the zero condition’ [35, 36]. A functional form that satisfied this requirement included the interaction between the FACT-8D levels and the *TIME* variable (Eq. 1). Therefore, as *TIME* tended to zero, the systematic component of the utility function tended to zero. Another typical requirement of the QALY model is constant proportional time trade-off [33], therefore a linear relationship between utility and *TIME* (life years) was assumed.

A useful feature of this functional form is that the impact of deviating from Level 1 (no problems) in each dimension was characterized through a two-factor interaction term with duration (the experimental design allowed for these interactions). This enabled a preference-weighting algorithm in which the effect of each level of each dimension could be included as a decrement from full health.

The analysis (Eq. 1) used conditional logit models in which the utility of option *j* in choice-set *s* for survey respondent *i* was assumed to be:

1 $$\begin{array}{c}{U}_{isj}=\alpha {TIME}_{isj}+ \beta {X}_{isj}{\prime}{TIME}_{isj}\\+{\varepsilon }_{isj} i = 1, \dots , I \mathrm{respondents}; j\\=\text{ health state options A},\text{ B}; s = 1, \dots , 800\text{ choice}-\mathrm{sets}\end{array}$$

where *α* is the utility associated with a life year,${X}_{isj}{\prime}$ is a vector of dummy variables representing the levels of the FACT-8D health state presented in option *j*, and *β* is the corresponding vector of preference weights associated with each level in each dimension within ${X}_{isj}{\prime}$, for each life year. The error term ${\varepsilon }_{ isj}$ was assumed to have a Gumbel distribution. To adjust the standard errors to allow for intra-individual correlation, a clustered sandwich estimator was used via STATA’s *vce* (cluster) option. To estimate preference weights for each deviation from Level 1 (no problems) in each FACT-8D dimension, we divided each of the *β* terms by *α* [32], and used the delta method to estimate standard errors for these ratios [37].

Two conditional logit models were estimated. Model 1 included every decrement from the best level (i.e., Level 1, no problems) in each dimension (Eq. 1). Thus, ${X}_{isj}{\prime}$ contained 32 terms (8 dimensions x [5-1] levels within each). Non-monotonicity in such models typically reflects noise, with the non-monotonic parameter estimates being not statistically different from each other [6]. Model 2 followed the same general form as Eq. 1 but imposed a restriction of monotonicity across levels of each dimension by combining adjacent non-monotonic levels. Model 2 therefore included a reduced number of estimates in *β* (the vector of preference weights). The MAUCa consortium has used this approach previously for the EORTC QLU-C10D [38–46]and the FACT-8D [10, 23]. The impact of constraining coefficients was assessed with change in the model pseudo R^2^; ideally, the imposition of monotonicity would not reduce model fit markedly.

For sociodemographic variables that deviated from the UK general population (using the latest available census data in England and Wales at time of analysis) by ≥ 2.0% in any category, iterative proportional fitting, or raking, weights were included in DCE models [47]. Raking was implemented using the *ipfweight* command in STATA 13.0, with observations with missing demographic data assigned a weight of one. Variance inflation due to weighting was assessed by calculating the percentage increase in the standard errors of the unweighted versus weighted Model 1 coefficients.

## Results

### Sample completion rates and representativeness

Survey completion rates are summarized in Fig. 2. Of the 3245 panel members who entered the survey, 462 (14.2%) either refused consent or did not respond further and 544 (16.8%) were excluded because sampling quotas were met. Of the remaining 2239, 2125 (95%) completed at least one or more choice sets, and 2054 (92%) completed all 16 choice sets. The data from these 2054 study participants were included in analyses to assess representativeness and to estimate the FACT-UK utility set.

The socio-demographic characteristics of the DCE analysis sample are compared to those of the general population in Table 2. The sample was representative of the general UK population for the quota sampling variables, age (*p* = 0.99) and sex (*p* = 0.95), but a greater proportion was born in England (79% vs 72%) and fewer were born in a non-English speaking country (6% vs 11%). The sample was more highly educated than the general population (*p* < 0.0001), with marked differences at the lowest (13% vs 37%) and highest (51% vs 27%) levels of education. Differences in marital status were relatively small (< = 3.2%) but statistically significant (*p* < 0.001). A higher proportion of the study sample owned/part owned a house (71% vs 65%) and fewer rented (27% vs 32%). Thirty-nine percent of the study sample reported excellent or very good health, 33% reported good health and 28% reported fair or poor health; there were no comparative data from the UK general population for the general health question. Four variables deviated from the general population by ≥ 2.0% in at least one category: country of birth, education, marital status and household ownership. These were included as raking variables in the DCE analyses.

**Table 2:** Self-reported sociodemographic characteristics and health of the UK valuation survey (n = 2054 participants who completed the 16 Discrete Choice Experiment choice sets) compared with the UK general population

Characteristics	Category	Sample, n	Sample, % or mean,$\overline{x }$	UK population, %	*p* value^a^
Sex	Male	999	48.6%	48.7%	.954
	Female	1055	51.4%	51.3%	
Age (years)	18–29	411	20.0%	20.1%	.999
	30–39	337	16.4%	16.5%	
	40–49	360	17.5%	17.4%	
	50–59	342	16.7%	16.6%	
	60–69	283	13.8%	13.9%	
	70 or older	321	15.6%	15.6%	
Country of birth	England	1604	78.6%	71.9%	<.0001
	Other UK^b^	268	13.1%	15.5%	
	Republic of Ireland plus other English‐speaking country	56	2.7%	1.50%	
	Other non‐English‐speaking country	113	5.5%	11.1%	
	Missing	13	-		
Highest level of education	Level 1^c^ or lower	260	12.7%	37.3%	<.0001
	Level 2^d^	313	15.3%	18.5%	
	Level 3^e^	324	15.9%	12.1%	
	Level 4^f^	1030	50.5%	27.0%	
	Other^g^	114	5.6%	5.1%	
	Missing	13	-		
Marital status	Single (never married or never registered a same‐sex civil partnership)	722	35.4%	34.7%	<.0001
	Married/in a registered same-sex civil partnership	1007	49.3%	46.7%	
	Other^h^	312	15.3%	18.5%	
	Missing	13	-		
Household ownership	Owns outright/owns with a mortgage or loan/part owns and part rents	1442	71.0%	65.0%	< 0.0001
	Rents	553	27.0%	32.0%	
	Lives rent free	46	2.0%	3.0%	
	Missing	13	-		
General Health Question^i^	Excellent [5]	209	10.2%	-	
	Very good [4]	594	28.9%	-	
	Good [3]	676	32.9%	-	
	Fair [2]	449	21.9%	-	
	Poor [1]	126	6.1%	-	
Total sample	2054				

Population values obtained from the 2011 U.K. census, available at https://www.ons.gov.uk/census#censusdataandbackground

^a^p values of <.05 suggest that sample is not representative of the general population. The chi‐squared goodness‐of‐fit test was used to compare observed category frequencies with those expected based on population proportions

^b^Other UK includes Scotland, Wales and Northern Ireland

^c^Level 1 is defined as 1–4 O levels/CSEs/GCSEs (any grades), entry level, foundation diploma, and NVQ Level 1, foundation GNVQ, basic skills

^d^Level 2 is defined as 5 + O levels (passes)/CSEs (Grade 1)/GCSEs (Grades A*–C), school certificate, 1 A level/2–3 AS levels/VCEs, higher diploma and NVQ Level 2, intermediate GNVQ, city and guilds craft, BTEC first/general diploma, RSA diploma, Apprenticeship

^e^Level 3 is defined as 2 + A levels/VCEs, 4 + AS levels, higher school certificate, progression/advanced diploma and NVQ Level 3, advanced GNVQ, city and guilds advanced craft, ONC, OND, BTEC national, RSA advanced diploma

^f^Level 4 + is defined as degree (for example BA, BSc), higher Degree (for example MA, PhD, PGCE), NVQ Level 4–5, HNC, HND, RSA Higher Diploma, BTEC Higher level, professional qualifications (for example teaching, nursing, accountancy)

^g^Other includes all participants who selected the “other vocational/work-related qualifications” or “foreign qualification” options

^h^Other includes separated (but still legally married or still legally in a same‐sex civil partnership); divorced or formerly in a same‐sex civil partnership which is now legally dissolved; and, widowed or surviving partner from a same‐sex civil partnership

^i^ The question asked was ‘In general would you say that your health is’, with five response options shown in this row

### Data quality metrics

The median time to complete the survey was 13.1 min (interquartile range 9.3–18.7 min). The plots of model fit and number of statistically significant coefficients by completion time decile showed that the fastest 20% of completers had the poorest model fit and least number of statistically significant coefficients (supplementary Fig. A). We considered excluding these respondents, based the value set was very similar after the exclusion. Since time to complete is a very imperfect measure of engagement and data quality, we therefore decided to retain all data in the analysis. Respondent feedback on the DCE task is provided in supplementary Appendix A. Briefly, of the 2053 participants who answered questions around the survey itself, 41% rated the difficulty of this survey as ‘about the same’ compared with other surveys they had done, while 45% rated it as ‘harder’. Most participants (86%) rated the presentation of the health states as ‘clear’ or ‘very clear’. While 45% of participants found it ‘very difficult’ or ‘difficult’ to choose between pairs of health states, 26% found it ‘easy’ or ‘very easy’.

### Preference modelling

**Fig. 3:**
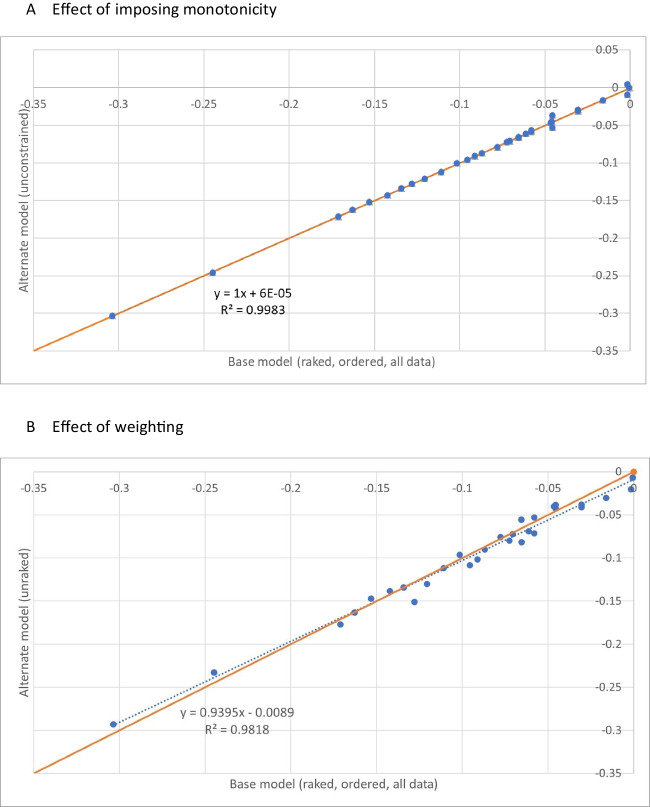
Scatterplots showing the effect of imposing monotonicity (panel **A**) and weighting (panel **B**) on preference weight estimates, relative to the weighted, ordered base model, showing line of best fit (dotted) and line of equality (solid)

Estimates of the coefficients in the four pre-specified conditional logit models ((Model 1-unconstrained and Model 2-monotonicity imposed) x (unweighted and weighted)) are reported in supplementary Table B. Imposing monotonicity (‘ordering’) and weighting generally had little effect on preference weight estimates (Fig. 3). Across all four models, duration had a large and positive coefficient, showing that study participants generally valued longer life. The HRQL coefficients were all negative. The HRQL dimensions with the largest decrements were pain and nausea, suggesting they had the greatest effect on respondents’ preferences for FACT-8D health states. The remaining dimensions had more moderate impacts on preferences.

In Model 1, one non-monotonic dimension was observed in the unweighted analysis and four dimensions had non-monotonicity when weighted (marked in italics in Table B), all for coefficients of levels 2 and 3 interacted with duration. Re-estimation with these disordered levels combined (Model 2) reduced the pseudo R^2^ very marginally in both unweighted and weighted analyses. Standard errors of Model 1 coefficients in weighted analyses were on average 29% larger than in unweighted analyses.

**Fig. 4:**
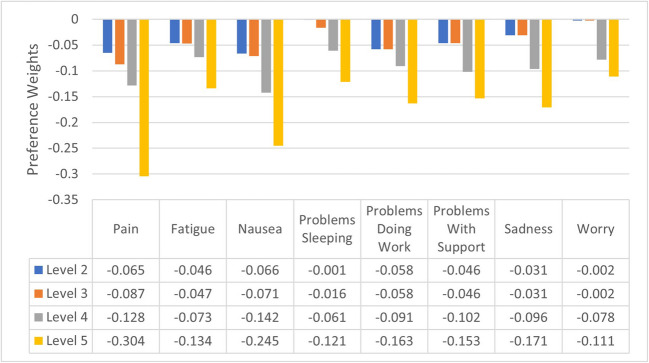
The UK FACT-8D value set

For the purposes of economic evaluation in consideration of the criteria to select a preferred model, we recommend using the preference weights derived from the weighted constrained conditional logit model; these reflect the views of a representative weighted sample of the general population and ensure the monotonic nature of the FACT-8D dimensions are reflected in the UK scoring algorithm. These are presented in Fig. 4, supplementary Table C, and in the scoring instructions (supplementary Appendix B) and corresponding code for use in STATA/SPSS software (supplementary Appendix C). To illustrate this scoring, if a patient’s FACT-G responses indicate that patient is at level 1 for all FACT-8D dimensions other than fatigue and worry where they are at level 3, their health state would be valued at 1- 0.047 - 0.002 = 0.951. The utility value of the worst health state is −0.402.

## Discussion

This study provided a value set representing the preferences of the UK general population for health states described by the FACT-8D, a MAUI derived from the FACT-G, a widely-used cancer-specific HRQL questionnaire [12]. Of the dimensions included, pain and nausea (which are common symptoms of cancer) had the largest decrements in the FACT-8D value set, followed by problems with social support, work and sadness. The FACT-8D scoring function enables direct calculation (i.e. a non-mapping approach) of UK-specific health utilities from FACT-G data. The FACT-8D has broad applicability, as many FACIT questionnaires contain the FACT-G items. Given the widespread use of the FACIT questionnaires in the UK and in global studies that support decision making in the UK, this algorithm has potential, in the same way as the corresponding UK algorithm for the EORTC QLU-C10D [45], to facilitate incorporation of common symptoms and impacts of cancer into the wide range of HTA conducted in the region. Recent work by Gamper et al. discussed the landscape in which the FACT-8D operates, identifying situations the current processes which might increase its use, and the specific advantages of doing so [48]. Of particular importance is the work of Gibson et al., who demonstrate through semi-structured interviews of patients receiving drug treatment for cancer that condition-specific measures (specifically the QLU-C10D and FACT-8D) had better relevancy than generic PBMs [49]. The importance of nausea in the value set emphasises this point; nausea is not explicitly part of any generic PBM to our knowledge, and is also a common side effect of many oncology therapies. Hence, using the FACT-8D over a generic PBM is likely to better capture health and changes in health in cancer settings where nausea is a common feature of treatment.

**Fig. 5:**
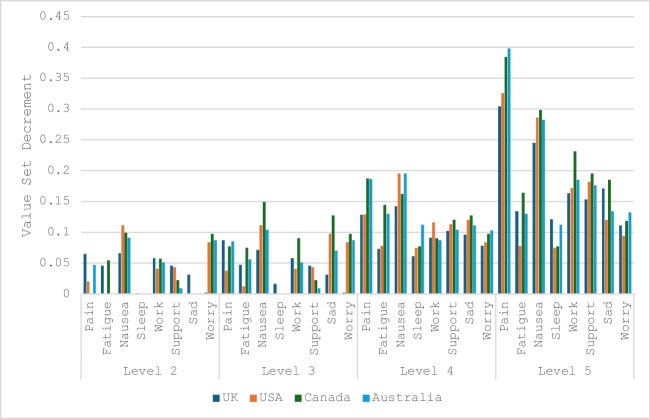
FACT-8D value set comparison

Adding this new value set to HTA toolkits prompts several questions, including how and why value sets differ and whether that difference reflects a significant issue. Factors that can affect health state values include the valuation method (e.g. time trade-off or DCE, plus specific method details), the health states being valued by respondents (the kind and number of dimensions, items and levels), and the survey respondent population (e.g. general population versus patients, different nationalities and health and sociodemographic profiles). Existing FACT-8D valuation studies have used the same protocol for data collection, equivalent utility modelling approaches in general population samples, and have used quota sampling and raking to achieve population-representativeness. These methodological consistencies are useful in that they give relatively well-controlled comparisons. The UK FACT-8D preference weights reported here were generally similar to those from Australia, the USA, and Canada; these similarities reiterate that when the same health states are valued with the same method in countries with similar cultures, similar results tend to ensue. A comparison of value sets is provided in Fig. 5. The minimum value under each of the values sets ranges from −0.652 (Canada) to −0.402 (UK).This echoes results from the EuroQol group, which found similar health-state values for the EQ-5D-5L across England, Canada, the Netherlands and the United States, using a common DCE (without survival) valuation protocol [50].

Head-to-head comparisons of the FACT-8D with generic MAUIs such as EQ-5D-5L will inform the debate over the ability of generic and disease-specific instruments to capture treatment effects [8, 51]. Existing studies in the area include one exploring performance of instruments from a randomised trial of 250 patients with non-Hodgkin’s lymphoma completing the disease-specific FACT-Lym and the EQ-5D-5L [52]. It found that both the FACT-8D and EQ-5D-5L showed good convergent validity and similar responsiveness to change, but that the EQ-5D-5L performed relatively better in terms of known groups’ validity. Similar analyses in other cancer types and treatments are needed to evaluate relative value and merits of FACT-8D versus PBMs that are not cancer-specific. However, such psychometric analyses have limited interpretation for the use of cancer-specific versus generic MAUIs in cost-utility analyses, which is potentially better achieved by head-to-head comparisons of the estimates of resultant QALYs in cost-utility analyses, as illustrated in comparisons of the EORTC QLU-C10 with the EQ-5D [53–55].

The FACT-8D allows prediction of utilities without the need for collection of another health preference measure. The direct generation of utilities using the FACT-8D, which contains cancer-specific items that are important to the patient, is preferable to mapping to another measure to generate utilities, which is always a second best solution to the use of a MAUI directly [56, 57]. However, there are potential barriers to its use as a primary preference-based measure in HTA in the UK because there remains considerable value and support for using generic instruments, which seek to quantify health improvements across conditions in an equal way. When designing studies, choice of measure should be matched to the patient population, intervention and relevant regulatory agencies’ HTA requirements as well as considerations of respondent burden. Depending on context, even when a generic measure is preferred for the reference case, the FACT-8D could serve as an adjunct measure to improve robustness of evidence in HTA, and can have an important role in sensitivity analyses [58].

This study had strengths and limitations. We used a DCE approach previously established as feasible [10, 21] and modelling approaches appropriate to our data structure and analysis purpose [34]. We simplified our DCE choice task by not asking respondents to trade across all dimensions at once, so the experimental design was not strictly orthogonal; we felt this was the right balance between statistical and respondent efficiency, given the cognitive complexity of the task. The FACT-8D DCE presented a challenge: when we piloted the DCE with the FACT-G’s verbatim positive framing of the work, sleep, and support items, credible preference weights could not be derived [10]. We solved this by reversing the polarity of these items, as discussed elsewhere [10, 23]. Our approach to DCE modelling, including constraining coefficients to ensure monotonicity, has been used across all MAUCa studies for calculating utility is readily accessible for a range of end-users, clinically interpretable and consistent with the FACIT conceptual model. Patterns of the time taken to complete the DCE task suggested most respondents engaged with the valuation task. The valuation survey sample was large, with quota sampling achieving population representativeness for age and sex. However, recruitment was restricted to participants who were able to read English, which may have introduced some bias. Further, the survey was conducted during the COVID-19 pandemic which had substantial impact on life in the UK. The pandemic did not impact on the ability or conduct online surveys such as this one during the pandemic, and indeed greater use and development of online research occurred during the pandemic [59], as well as increased participation in online activities amongst the UK population. However, it is unknown whether there was an impact on health preferences during the pandemic for the health attributes assessed here in comparison to pre and post-pandemic preferences. There is limited evidence about the impacts of the COVID-19 pandemic on how people value health, and the policy implications of any such effects are unclear [60]. We assessed population representativeness across a range of demographic characteristics and adjusted for non-representativeness using raking, which has benefits in terms of bringing the sample in line with pre-specified population characteristics, but costs in terms of inflating variances, particularly when the sample and population differ by a large amount [47]. Even though the standard errors of weighted model coefficients were inflated, in only two cases did this change the level of statistical significance, and the preference weights derived using raked or unraked responses were very similar. The influence of DCE respondent sociodemographic characteristics on preference weights will be assessed in future analysis of pooled data from international valuations of the FACT-8D. Non-representativeness in unmeasured variables is, as with any survey, a covert threat to generalizability, which is difficult to mitigate. Although online panels provide convenient economical sampling frames and represent a popular sampling choice in modern science, potential selection biases deserve further research [61].

## Conclusions

The UK FACT-8D utility algorithm provides a new option for obtaining utility values and QALYs from oncology trials that have used the FACT-G or other FACIT questionnaires in which it is embedded. Further evidence is needed of the FACT-8D’s performance relative to generic utility measures across a range of clinical settings, and until this evidence is available it is recommended for use alongside an accepted generic measure.

## Supplementary Information

Below is the link to the electronic supplementary material.

Supplementary file1 (JPG 217 KB)

Supplementary file2 (DOCX 20 KB)

Supplementary file3 (DOCX 16 KB)

Supplementary file4 (DOCX 28 KB)

Supplementary file5 (DOCX 27 KB)

Supplementary file6 (DOCX 22 KB)

## Data Availability

Data are available from the authors on reasonable request, and subject to the constraints of the project's ethics approval.

## Declarations

### Concise description

Provides the United Kingdom value set for the cancer-specific FACT-8D preference-based quality of life instrument, derived from the FACT-G health-related quality of life questionnaire.
