# Supplementary figures and images for: United Kingdom value set for the functional assessment of cancer therapy eight dimension (FACT-8D) preference-based quality of life instrument
Source: Eur J Health Econ. 2025 Oct 8;27(3):609–22. doi: 10.1007/s10198-025-01844-w (PMC13190361; doi:10.1007/s10198-025-01844-w)

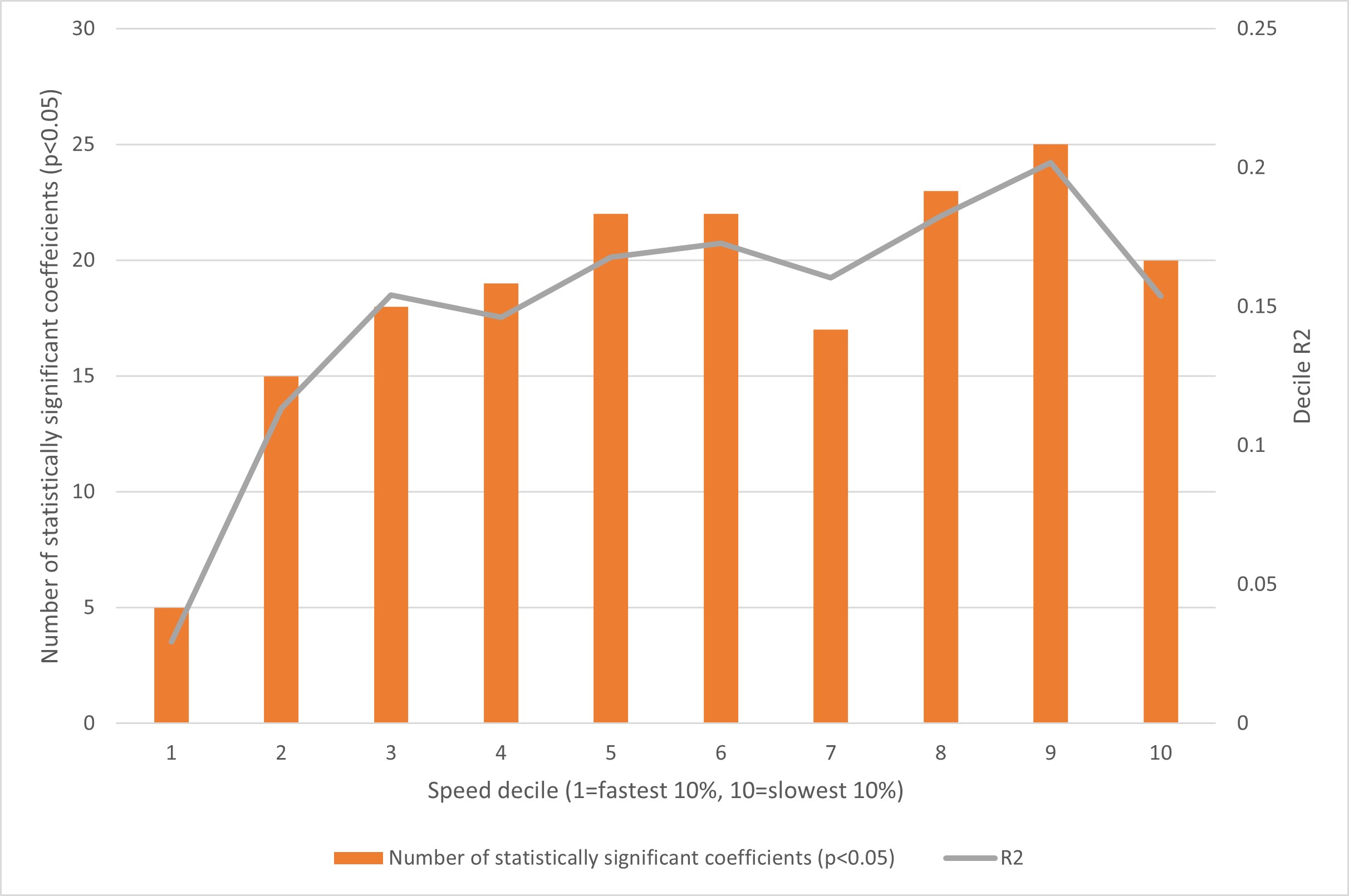

Supplement: Supplementary file 1 — Supplementary file1 (JPG 217 KB) [file 10198_2025_1844_MOESM1_ESM.jpg]
