# Supplementary material for: United Kingdom value set for the functional assessment of cancer therapy eight dimension (FACT-8D) preference-based quality of life instrument
Source: Eur J Health Econ. 2025 Oct 8;27(3):609–22. doi: 10.1007/s10198-025-01844-w (PMC13190361; doi:10.1007/s10198-025-01844-w)
Supplement: Supplementary file 2 — Supplementary file2 (DOCX 20 KB) [file 10198_2025_1844_MOESM2_ESM.docx]

**Table B** Conditional logit results for Model 1 (unconstrained) and Model 2 (monotonicity imposed^a^, ordered), unweighted and weighted analyses (estimated coefficients and robust standard errors (SE))

| Coefficient (SE) |  | Unweighted Analyses | | Weighted Analyses | |
| --- | --- | --- | --- | --- | --- |
| Dimension | Level | Unconstrained | Constrained | Unconstrained | Constrained |
| Duration | Linear (years) | 0.546 (0.018)*** | 0.546 (0.018)*** | 0.504 (0.024)*** | 0.504 (0.024)*** |
| Duration x Pain | Level 2 | -0.045 (0.007)*** | -0.045 (0.007)*** | -0.033 (0.009)*** | -0.033 (0.009)*** |
|  | Level 3 | -0.049 (0.007)*** | -0.049 (0.007)*** | -0.044 (0.01)*** | -0.044 (0.01)*** |
|  | Level 4 | -0.082 (0.008)*** | -0.082 (0.008)*** | -0.064 (0.01)*** | -0.065 (0.01)*** |
|  | Level 5 | -0.160 (0.008)*** | -0.160 (0.008)*** | -0.153 (0.01)*** | -0.153 (0.01)*** |
| Duration x Fatigue | Level 2 | -0.021 (0.006)*** | -0.021 (0.006)*** | -0.023 (0.008)*** | -0.023 (0.008)*** |
|  | Level 3 | -0.022 (0.007)*** | -0.022 (0.007)*** | -0.023 (0.009)*** | -0.024 (0.009)*** |
|  | Level 4 | -0.044 (0.007)*** | -0.044 (0.007)*** | -0.036 (0.009)*** | -0.037 (0.009)*** |
|  | Level 5 | -0.073 (0.007)*** | -0.073 (0.007)*** | -0.067 (0.009)*** | -0.068 (0.009)*** |
| Duration x Nausea | Level 2 | -0.030 (0.005)*** | -0.030 (0.005)*** | -0.033 (0.007)*** | -0.033 (0.007)*** |
|  | Level 3 | -0.039 (0.006)*** | -0.039 (0.006)*** | -0.036 (0.007)*** | -0.036 (0.007)*** |
|  | Level 4 | -0.076 (0.006)*** | -0.076 (0.006)*** | -0.072 (0.008)*** | -0.072 (0.008)*** |
|  | Level 5 | -0.127 (0.006)*** | -0.127 (0.006)*** | -0.124 (0.009)*** | -0.123 (0.008)*** |
| Duration x Problems Sleeping | Level 2 | -0.004 (0.007) | -0.004 (0.007) | 0 (0.009) | 0 (0.009) |
|  | Level 3 | -0.017 (0.006)*** | -0.017 (0.006)*** | -0.008 (0.009) | -0.008 (0.009) |
|  | Level 4 | -0.038 (0.006)*** | -0.038 (0.006)*** | -0.031 (0.009)*** | -0.031 (0.009)*** |
|  | Level 5 | -0.071 (0.007)*** | -0.071 (0.007)*** | -0.061 (0.009)*** | -0.061 (0.009)*** |
| Duration x Problems Working | Level 2 | -0.029 (0.005)*** | -0.029 (0.005)*** | -0.030 (0.007)*** | *-0.029 (0.006)**** |
|  | Level 3 | -0.039 (0.005)*** | -0.039 (0.005)*** | -0.028 (0.007)*** | *-0.029 (0.006)**** |
|  | Level 4 | -0.056 (0.005)*** | -0.056 (0.005)*** | -0.046 (0.007)*** | -0.046 (0.007)*** |
|  | Level 5 | -0.089 (0.006)*** | -0.089 (0.006)*** | -0.082 (0.008)*** | -0.082 (0.008)*** |
| Duration x Problems with Support | Level 2 | -0.021 (0.006)*** | -0.021 (0.006)*** | -0.027 (0.008)*** | *-0.023 (0.007)**** |
|  | Level 3 | -0.023 (0.007)*** | -0.023 (0.007)*** | -0.019 (0.009)** | *-0.023 (0.007)**** |
|  | Level 4 | -0.053 (0.007)*** | -0.053 (0.007)*** | -0.050 (0.009)*** | -0.051 (0.008)*** |
|  | Level 5 | -0.080 (0.007)*** | -0.080 (0.007)*** | -0.077 (0.009)*** | -0.077 (0.009)*** |
| Duration x Sadness | Level 2 | -0.021 (0.007)*** | -0.021 (0.007)*** | -0.016 (0.009)* | *-0.015 (0.007)*** |
|  | Level 3 | -0.022 (0.006)*** | -0.022 (0.006)*** | -0.015 (0.008)* | *-0.015 (0.007)*** |
|  | Level 4 | -0.059 (0.007)*** | -0.059 (0.007)*** | -0.048 (0.009)*** | -0.048 (0.009)*** |
|  | Level 5 | -0.097 (0.007)*** | -0.097 (0.007)*** | -0.086 (0.009)*** | -0.086 (0.009)*** |
| Duration x Worry | Level 2 | -0.011 (0.007)* | *-0.011 (0.005)*** | -0.005 (0.009) | *-0.001 (0.007)* |
|  | Level 3 | -0.011 (0.006)* | *-0.011 (0.005)*** | 0.002 (0.009) | *-0.001 (0.007)* |
|  | Level 4 | -0.041 (0.006)*** | -0.041 (0.006)*** | -0.040 (0.008)*** | -0.039 (0.008)*** |
|  | Level 5 | -0.061 (0.007)*** | -0.061 (0.007)*** | -0.056 (0.009)*** | -0.056 (0.009)*** |
| Pseudo R2 | | 0.1353 | 0.1353 | 0.1295 | 0.1294 |
| Log Pseudolikelihood | | -19923 | -19923 | -20045 | -20047 |
| AIC | | 39911 | 39909 | 40156 | 40151 |
| BIC | | 40212 | 40201 | 40457 | 40415 |

1. The coefficient for each level of each QOL dimension was estimated as the interaction of that level with duration. *Levels combined to ensure monotonicity within each dimension are noted in italics.*

Levels of statistical significance: ***1%; **5%; *10%
