# Supplementary material for: United Kingdom value set for the functional assessment of cancer therapy eight dimension (FACT-8D) preference-based quality of life instrument
Source: Eur J Health Econ. 2025 Oct 8;27(3):609–22. doi: 10.1007/s10198-025-01844-w (PMC13190361; doi:10.1007/s10198-025-01844-w)
Supplement: Supplementary file 3 — Supplementary file3 (DOCX 16 KB) [file 10198_2025_1844_MOESM3_ESM.docx]

**Table C** Preference weights in the UK FACT-8D utility algorithm

| FACT-8D Dimension  *DCE Attribute wording (if different)* | FACT-8D Level | Corresponding FACT-G score | Utility decrement  (95% CI) |
| --- | --- | --- | --- |
| Pain | 2 | 1 | 0.065 (0.032,0.098) |
|  | 3 | 2 | 0.087 (0.050,0.124) |
|  | 4 | 3 | 0.128 (0.092,0.163) |
|  | 5 | 4 | 0.304 (0.269,0.338) |
|  |  |  |  |
| Fatigue | 2 | 1 | 0.046 (0.015,0.076) |
|  | 3 | 2 | 0.047 (0.013,0.080) |
|  | 4 | 3 | 0.073 (0.039,0.106) |
|  | 5 | 4 | 0.134 (0.103,0.166) |
|  |  |  |  |
| Nausea | 2 | 1 | 0.066 (0.040,0.091) |
|  | 3 | 2 | 0.071 (0.043,0.098) |
|  | 4 | 3 | 0.142 (0.113,0.171) |
|  | 5 | 4 | 0.245 (0.213,0.277) |
|  |  |  |  |
| Sleep  *Problems sleeping* | 2 | 3 | 0.001 (-0.033,0.034) |
|  | 3 | 2 | 0.016 (-0.019,0.051) |
|  | 4 | 1 | 0.061 (0.029,0.093) |
|  | 5 | 0 | 0.121 (0.089,0.152) |
|  |  |  |  |
| Work  *Problems doing work (including work at home)* | 2/3 | 3/2 | 0.058 (0.035,0.081) |
|  | 4 | 1 | 0.091 (0.064,0.118) |
|  | 5 | 0 | 0.163 (0.135,0.191) |
|  |  |  |  |
| Support  *Problems with support from my family and/or friends* | 2/3 | 3/2 | 0.046 (0.018,0.074) |
|  | 4 | 1 | 0.102 (0.070,0.133) |
|  | 5 | 0 | 0.153 (0.122,0.184) |
|  |  |  |  |
| Sadness | 2/3 | 1/2 | 0.031 (0.002,0.059) |
|  | 4 | 3 | 0.096 (0.065,0.127) |
|  | 5 | 4 | 0.171 (0.138,0.204) |
|  |  |  |  |
| Worry my health will get worse | 2/3 | 1/2 | 0.002 (-0.027,0.030) |
|  | 4 | 3 | 0.078 (0.049,0.107) |
|  | 5 | 4 | 0.111 (0.075,0.147) |
