# Supplementary material for: United Kingdom value set for the functional assessment of cancer therapy eight dimension (FACT-8D) preference-based quality of life instrument
Source: Eur J Health Econ. 2025 Oct 8;27(3):609–22. doi: 10.1007/s10198-025-01844-w (PMC13190361; doi:10.1007/s10198-025-01844-w)
Supplement: Supplementary file 4 — Supplementary file4 (DOCX 28 KB) [file 10198_2025_1844_MOESM4_ESM.docx]

**Appendix A: Participant Feedback on the Discrete Choice Experiment (DCE) Valuation Task**

**Methods**

After completing the valuation task (16 DCE choice sets), participants were asked four fixed-format questions about the difficulty and clarity of the valuation task and which strategy they used to choose between the pairs of health states. For the question about strategy, participants who selected the ‘Other’ response option were invited to provide further detail in an open text field. The fixed-format questions asked and their response options are provided in Table 1 below.

**Results**

Of the 2053 participants who answered these questions, 41% rated the difficulty of this survey as ‘about the same’ compared with other surveys they had done, while 45% rated it as ‘harder’ (Table 1a). Most participants (86%) rated the presentation of the health states as ‘clear’ or ‘very clear’ (Table 1b). While 45% of participants found it ‘very difficult’ or ‘difficult’ to choose between pairs of health states, 26% found it ‘easy’ or ‘very easy’ (Table 1c). When asked about the strategy used by participants to choose between the pairs of health states, 24% indicated they considered all aspects of the health state, 19% reported focusing on the aspects highlighted in yellow, 25% said they focused on just a few aspects, and 8% indicated they did not use a strategy (Table 1d). The remaining 1% (n=20) reported using another strategy; 15 of these participants provided a brief free-text description of their strategy (Table 2). Among these, survival was the dominant theme (mentioned by n=8/15, 53%). Pain was noted as the key choice driver by 2 participants and a consideration by another two (n=4/15, 27%). Other symptoms and family relationship were also noted by some participants.

**Table 1.** Participants’ perceptions of the DCE task (a-c) and their choice strategies (d), n=2053.

| (a) Did you find the questions on these 16 screens easier or harder than most surveys you do? | | |
| --- | --- | --- |
| Response | **Frequency** | **Percent** |
| Easier | 269 | 13.1% |
| About the same | 836 | 40.7% |
| Harder | 922 | 44.9% |
| I couldn’t say | 26 | 1.3% |
| Total | **2053** | **100%** |
| (b) How clear was the presentation of the health states? | | |
| Response | **Frequency** | **Percent** |
| Very unclear | 33 | 1.6 % |
| Unclear | 80 | 3.9% |
| Neither clear nor unclear | 244 | 11.9% |
| Clear | 996 | 48.5% |
| Very clear | 700 | 34.1% |
| Total | **2053** | **100%** |
| (c) How difficult was it to choose between the pairs of health states on each screen? | | |
| Response | **Frequency** | **Percent** |
| Very difficult | 152 | 7.4 |
| Difficult | 776 | 37.8 |
| Neither easy nor difficult | 586 | 28.5 |
| Easy | 387 | 18.9 |
| Very easy | 152 | 7.4 |
| Total | **2053** | **100%** |
| (d) Did you have a strategy for choosing between the pairs of health states on each screen? | | |
| Response | **Frequency** | **Percent** |
| I did not have a strategy | 168 | 8.2% |
| I focused on just a few aspects of the health states | 514 | 25.0% |
| I focused on the aspects that were highlighted in yellow | 386 | 18.8% |
| I considered most of the aspects | 482 | 23.5% |
| I considered all of the aspects | 483 | 23.5% |
| Other* | 20 | 1.0% |
| Total | **2053** | 100% |

***** Participants who selected the response ‘Other’ could enter free text to describe the strategy they used. Of the 20 who selected ‘Other’, 15 entered informative text, while the remaining five entered non-sensical (e.g. ‘red I love’) or informative (e.g. ‘unsure’) text.

**Table 2** Coding of free text describing ‘Other’ strategy (n=15)

| Coding of free text describing ‘Other’ strategy | Frequency | Percent |
| --- | --- | --- |
| Length of survival | 5 | 33 |
| Length of survival and other aspects (1 noted pain specifically) | 3 | 20 |
| Considered all aspects (their free-text described their decision process) | 2 | 13 |
| All aspects except for length of survival, noting pain, sadness, fatigue, poor sleep and nausea specifically | 1 | 7 |
| Pain | 2 | 13 |
| Sleep | 1 | 7 |
| Family relationships | 1 | 7 |
| Total | **15** | **100** |
