# Supplementary material for: United Kingdom value set for the functional assessment of cancer therapy eight dimension (FACT-8D) preference-based quality of life instrument
Source: Eur J Health Econ. 2025 Oct 8;27(3):609–22. doi: 10.1007/s10198-025-01844-w (PMC13190361; doi:10.1007/s10198-025-01844-w)
Supplement: Supplementary file 5 — Supplementary file5 (DOCX 27 KB) [file 10198_2025_1844_MOESM5_ESM.docx]

**Appendix B: United Kingdom (UK) FACT-8D utility algorithm and scoring instructions**

This appendix contains the FACT-8D scoring algorithm and instructions for calculating UK-based FACT-8D utility scores (i.e. based on the stated preferences of the UK general population) from FACT-G responses.

The algorithm and instructions apply regardless of whether the FACT-G source data are collected with the stand-alone FACT-G questionnaire or any related FACIT questionnaire that has the FACT-G items embedded in it, and whether the FACT-G source data are collected in the UK or any other country.

**Abbreviations**

| FACT-8D | Functional Assessment of Cancer Therapy Eight Dimension |
| --- | --- |
| FACT-G | Functional Assessment of Cancer Therapy – General |
| FACIT | Functional Assessment of Chronic Illness Therapy |
| UK | United Kingdom |

**FACT-8D utility algorithm**

A utility score of 1 is assigned to individuals whose FACT-G scores indicate they are at the best level (Level 0) of all 8 dimensions of the FACT-8D. For all other health states, the utility score is 1 minus each preference weight (*w_dl_*) for each level down from the best level in each of the 8 FACT-8D dimensions.

$${FACT\text{-}8D}_{i}=1-\sum_{d=1}^{8} w_{dl}|{FACT\text{-}8D}_{dli}$$

**FACT-8D UK scoring instructions**

For any individual *i* who has provided responses to the nine FACT-G items that contribute to FACT-8D scores (see Table 1), the individual’s UK-based FACT-8D utility score is calculated as follows.

First, determine the corresponding level *l* for each FACT-8D dimension *d*, following the mapping of FACT-G item levels to FACT-8D dimension levels (see Table 1), and determine the associated preference weights (*w­_dl_*) estimated from the UK general population (see Table C).

Then subtract the sum of the preference weights from 1. This sum represents the total utility decrements across the 8 dimensions of the FACT-8D.

**Example**

A patient has completed the FACT-G questionnaire. Her responses indicate:

- *quite a bit of pain*,
- *somewhat lacking energy*,
- *no nausea*,
- *sleeping quite well*,
- *able to work a little bit*,
- *getting quite a bit of emotional support from family and some support from friend*s,
- *feeling a little bit sad*,
- *somewhat worried that her condition will get worse.*

This represents one of the many possible FACT-8D health state. The utility score for this health state is determined by mapping the patient’s FACT-G responses to the corresponding FACT-8D dimension levels, determining the corresponding preference weights, summing these to determine the total utility decrement for that health state, and substracting this sum from 1 (full health).

For the five negatively-framed FACT-8D dimensions, i.e. *Pain, Fatigue, Nausea, Sadness, Worry*

|  | Best possible |  |  |  | Worst possible |
| --- | --- | --- | --- | --- | --- |
| FACT-G response | 0  Not at all | 1  A little bit | 2  Somewhat | 3  Quite a bit | 4  Very much |
| FACT-8D level | 1 | 2 | 3 | 4 | 5 |

For the three positively-framed FACT-8D dimensions that require reverse scoring for FACT-8D, ie. *Sleep*, *Work*, *Support*, this is how it works:

|  | Best possible |  |  |  | Worst possible |
| --- | --- | --- | --- | --- | --- |
| FACT-G response  (positively framed) | 4  Very much | 3  Quite a bit | 2  Somewhat | 1  A little bit | 0  Not at all |
| FACT-8D level | 1 | 2 | 3 | 4 | 5 |

For Support: The FACT-8D Support dimension contains two items; the FACT-8D level allocated is the maximum score of the FACT-G items GS2 and GS3, i.e. the best level of support, whether from family or friends.

For this particular health state, this is the correct mapping, corresponding preference weights.

| FACT-G  Item | FACT-G  Response | FACT-8D Dimension | FACT-8D Dimension level | UK Preference Weight |
| --- | --- | --- | --- | --- |
| GP4: *I have pain* | *Quite a bit* = 3 | Pain | 4 | -0.128 |
| GP1: *I have a lack of energy* | *Somewhat* = 2 | Fatigue | 3 | -0.047 |
| GP2: *I have nausea* | *Not at all* = 0 | Nausea | 1 | 0 |
| GF5: *I am sleeping well* | *Quite a bit* = 3 | Sleep^b^ | 2 | 0 |
| GF1: *I am able to work* | *A little bit* = 1 | Work^b^ | 4 | -0.091 |
| GS2 *I get emotional support from my family*  GS3: *I get support from my friends* | *Quite a bit* = 3  *Somewhat* = 2 | Support^b^ | 2 | -0.046 |
| GE1: *I feel sad* | *A little bit* = 1 | Sadness | 2 | 0.031 |
| GE6: *I worry that my condition will get worse* | *Somewhat* = 2 | Worry my health will get worse | 3 | -0.002 |
| Total utility decrement | | | | -0.283 |

The valued of this health state is therefore 1 – 0.283 = 0.717

The best possible health state has a value of 1, and the worst possible health state (the ‘pits’) has a value of 1 minus the sum of all the Level 4 preference weights:

Value of the pits state = 1 – (0.304 + 0.134 + 0.245 + 0.121 + 0.163 + 0.153 + 0.171 + 0.111)

= 1 – (1.402)

= -0.402
