# Supplementary material for: United Kingdom value set for the functional assessment of cancer therapy eight dimension (FACT-8D) preference-based quality of life instrument
Source: Eur J Health Econ. 2025 Oct 8;27(3):609–22. doi: 10.1007/s10198-025-01844-w (PMC13190361; doi:10.1007/s10198-025-01844-w)
Supplement: Supplementary file 6 — Supplementary file6 (DOCX 22 KB) [file 10198_2025_1844_MOESM6_ESM.docx]

STATA code to calculate FACT-8D utility scores from FACT-G responses using

United Kingdom (UK)-based preference weights

Written by Richard Norman richard.norman@curtin.edu.au March 2023

* This code is designed to convert FACT-G responses into FACT-8D preference weights and calculate a utility score for each health in the input FACT-G data set.

* It uses the UK DCE-derived weights developed by [removed to prevent

* unblinding during peer-review].

* It is based on the assumption that the FACT-G underlying data are

* coded between 0 and 4

* where 0 means 'Not at all', 1 means 'A little bit', 2 means 'Somewhat',

* 3 means 'Quite a bit', and 4 means 'Very much'.

* The coding of the variables is clustered by domain,

* so Physical Well-Being items are labelled GP1-GP7,

* Social / Family Well-Being items are labelled GS1-GS7,

* Emotional Well-Being items are labelled GE1-GE6, and

* Functional Well-Being are labelled GF1-GF7.

*

gen pai = gp4

gen fat = gp1

gen nau = gp2

gen sad = ge1

gen wor = ge6

*Sleep, Work and Support dimensions are reverse coded

gen sle = 4-gf5

gen wrk = 4-gf1

gen sup = 4 - max(gs2,gs3)

gen paidec=.

replace paidec=0 if pai==0

replace paidec=-0.065 if pai==1

replace paidec=-0.087 if pai==2

replace paidec=-0.128 if pai==3

replace paidec=-0.304 if pai==4

gen fatdec=.

replace fatdec=0 if fat==0

replace fatdec=-0.046 if fat==1

replace fatdec=-0.047 if fat==2

replace fatdec=-0.073 if fat==3

replace fatdec=-0.134 if fat==4

gen naudec=.

replace naudec=0 if nau==0

replace naudec=-0.066 if nau==1

replace naudec=-0.071 if nau==2

replace naudec=-0.142 if nau==3

replace naudec=-0.245 if nau==4

gen sledec=.

replace sledec=0 if sle==0

replace sledec=-0.001 if sle==1

replace sledec=-0.016 if sle==2

replace sledec=-0.061 if sle==3

replace sledec=-0.121 if sle==4

gen wrkdec=.

replace wrkdec=0 if wrk==0

replace wrkdec=-0.058 if wrk==1

replace wrkdec=-0.058 if wrk==2

replace wrkdec=-0.091 if wrk==3

replace wrkdec=-0.163 if wrk==4

gen supdec=.

replace supdec=0 if sup==0

replace supdec=-0.046 if sup==1

replace supdec=-0.046 if sup==2

replace supdec=-0.102 if sup==3

replace supdec=-0.153 if sup==4

gen saddec=.

replace saddec=0 if sad==0

replace saddec=-0.031 if sad==1

replace saddec=-0.031 if sad==2

replace saddec=-0.096 if sad==3

replace saddec=-0.171 if sad==4

gen wordec=.

replace wordec=0 if wor==0

replace wordec=-0.002 if wor==1

replace wordec=-0.002 if wor==2

replace wordec=-0.078 if wor==3

replace wordec=-0.111 if wor==4

gen fact8d = 1 + paidec + fatdec + naudec + sledec + wrkdec + supdec + saddec + wordec

SPSS syntax

SPSS code to calculate FACT-8D utility scores from FACT-G responses using

United Kingdom(UK)-based preference weights

Written by Daniel Costa daniel.costa@sydney.edu.au 16th December 2020, and adapted by Rachel Campbell January 2023

* Encoding: UTF-8.

* Encoding: .

* This code is designed to convert FACT-G responses into FACT-8D preference weights and calculate a utility score for each health in the input FACT-G data set.

* It uses US DCE-derived weights developed by [removed to prevent

* unblinding during peer-review].

* It is based on the assumption that the underlying FACT-G data are coded

* between 0 and 4

* where 0 means 'Not at all', 1 means 'A little bit', 2 means 'Somewhat',

* 3 means 'Quite a bit', and 4 means 'Very much'.

* The coding of the variables is clustered by domain,

* so Physical Well-Being items are labelled GP1-GP7,

* Social / Family Well-Being items are labelled GS1-GS7,

* Emotional Well-Being items are labelled GE1-GE6, and

*Functional Well-Being are labelled GF1-GF7.

compute pai = gp4.

compute fat = gp1.

compute nau = gp2.

compute sad = ge1.

compute wor = ge6.

*Sleep, Work and Support dimensions are reverse coded

compute sle = 4-gf5.

compute wrk = 4-gf1.

compute sup = 4 - max(gs2,gs3).

exe.

compute paidec=$sysmis.

if pai=0 paidec=0.

if pai=1 paidec=-0.065.

if pai=2 paidec=-0.087.

if pai=3 paidec=-0.128.

if pai=4 paidec=-0.304.

compute fatdec= $sysmis.

if fat=0 fatdec=0.

if fat=1 fatdec=-0.046.

if fat=2 fatdec=-0.047.

if fat=3 fatdec=-0.073.

if fat=4 fatdec=-0.134.

compute naudec=$sysmis.

if nau=0 naudec=0.

if nau=1 naudec=-0.066.

if nau=2 naudec=-0.071.

if nau=3 naudec=-0.142.

if nau=4 naudec=-0.245.

compute sledec=$sysmis.

if sle=0 sledec=0.

if sle=1 sledec=-0.001.

if sle=2 sledec=-0.016.

if sle=3 sledec=-0.061.

if sle=4 sledec=-0.121.

compute wrkdec=$sysmis.

if wrk=0 wrkdec=0.

if wrk=1 wrkdec=-0.058.

if wrk=2 wrkdec=-0.058.

if wrk=3 wrkdec=-0.091.

if wrk=4 wrkdec=-0.163.

compute supdec=$sysmis.

if sup=0 supdec=0.

if sup=1 supdec=-0.046.

if sup=2 supdec=-0.046.

if sup=3 supdec=-0.102.

if sup=4 supdec=-0.153.

compute saddec=$sysmis.

if sad=0 saddec=0.

if sad=1 saddec=-0.031.

if sad=2 saddec=-0.031.

if sad=3 saddec=-0.096.

if sad=4 saddec=-0.171.

compute wordec=$sysmis.

if wor=0 wordec=0.

if wor=1 wordec=-0.002.

if wor=2 wordec=-0.002.

if wor=3 wordec=-0.078.

if wor=4 wordec=-0.111.

compute fact8d = 1 + paidec + fatdec + naudec + sledec + wrkdec + supdec + saddec + wordec.

exe.
